# Gliomatosis Cerebri: Current Understanding and Controversies

**DOI:** 10.3389/fonc.2017.00165

**Published:** 2017-08-07

**Authors:** Surabhi Ranjan, Katherine E. Warren

**Affiliations:** ^1^Neuro-Oncology Branch, National Cancer Institute, National Institutes of Health, Bethesda, MD, United States; ^2^Pediatric-Oncology Branch, National Cancer Institute, National Institutes of Health, Bethesda, MD, United States

**Keywords:** gliomatosis cerebri, glioma, primary gliomatosis cerebri, secondary gliomatosis cerebri, review

## Abstract

Gliomatosis cerebri (GC) is a rare, extensively infiltrating glioma involving multiple contiguous lobes of the brain. This lethal disease affects all age groups, and the majority of patients have a poor outcome despite aggressive treatment. Despite its initial recognition in 1938, GC remains a controversial entity with little consensus in its definition, histology, or treatment. The majority of GC tumors are astrocytic, although mixed phenotypes have been identified. Treatment of GC is challenging as surgery is generally not an option due to the extensive areas of brain involved, the benefit of radiation therapy is unclear, and no chemotherapy has proven efficacy. Due to the rarity of the disease and its heterogeneity, both at histopathological and molecular levels, it is difficult to conduct clinical trials tailored for this diagnosis. This review summarizes our current knowledge, examines clinical studies focusing on the treatment of GC, highlights ongoing challenges, and discusses the recent molecular insights into adult and pediatric GC. We conclude that, although no longer recognized as a distinct pathological entity, GC represents a unique disease phenotype. Given the histologic and molecular overlap with other diffuse gliomas, the research emphasis should be on investigating its unique invasive biology.

## Introduction

Gliomatosis cerebri (GC) is a rare, diffusely infiltrating glial brain tumor. Prognosis is poor, with 26–52% surviving less than a year from symptom onset (1, 2). GC is a controversial disease entity as no consensus exists regarding its definition, histopathology, and treatment. The 2007 World Health Organization (WHO) classification of central nervous system (CNS) tumors defined GC as an extensively infiltrative diffuse glioma involving at least three cerebral lobes; however, the revised 2016 WHO CNS classification no longer identifies GC as a distinct *pathological* entity (3). Rather, this new classification designates it as a special pattern of growth and categorizes it under the various subtypes of diffuse gliomas. While this approach is legitimate within the scope of a pure histopathological classification, it implies that GC is simply a grand manifestation of diffuse glioma. Patients with GC generally have a worse prognosis compared to patients with diffuse glioma of corresponding grades. It is unclear whether this is due to the distinct GC biology causing extensive invasiveness or merely because of the fact the due to the large areas of brain involved, treatment options are limited. While progress has been made in the biological understanding and treatment of various gliomas, little progress has been made in understanding GC. The rarity of the disease, lack of in-depth understanding of the tumor biology, variation across histopathological grading, variability in patient outcomes, and the lack of durable response to therapies are major obstacles toward establishing standard treatments. This review highlights our current understanding of GC and discusses recent molecular diagnostics which may help in tailoring more efficacious therapeutic regimens. The question as to whether GC is a distinct disease entity or a distinctive phenotype of diffuse glioma will require additional investigations.

The histological, clinical, and radiographic classification of GC is not universally agreed upon. Classification systems are cumbersome and their clinical utility unclear. Conventionally, GC has been classified as primary or secondary GC (4, 5). Primary GC arises *de novo* and is further subclassified as type I (classic) when no obvious mass is present, or type II, where a diffuse infiltrative pattern coexists with an associated tumor mass (2). Secondary GC is defined as an infiltrative spread of tumor cells from a previously diagnosed glioma and is frequently associated with prior radiation or antiangiogenic therapy (2, 4). In light of the 2016 WHO classification update, GC is further subcategorized according to histopathologic grade and molecular findings, i.e., GC—diffuse astrocytoma, IDH-mutant; GC—diffuse astrocytoma, IDH-wildtype; GC—anaplastic astrocytoma, IDH-mutant; GC—anaplastic astrocytoma, IDH-wildtype; GC—glioblastoma, IDH-mutant; GC—glioblastoma, IDH-wildtype; GC—oligodendroglioma, IDH-mutant and 1p/19q-codeleted; GC—anaplastic oligodendroglioma, and IDH-mutant and 1p/19q-codeleted.

## Diagnosis

Gliomatosis cerebri spans across all age groups but is more common in adults. The median age at diagnosis ranges from 46 to 53 years (1, 6, 7) with a slight male predominance (sex ratio, 1.4) (6). Clinical presentation is variable and typically insidious, often delaying the diagnosis by months or years. Common presenting symptoms may be location dependent and include focal weakness, sensory loss, seizure, progressive headache or manifestations of increased intracranial pressure, memory deficit with “dementia-like” features, and other constitutional symptoms (2, 4, 5, 8). Common clinical signs include corticospinal tract, spinocerebellar, sensory-motor and visual field deficits, cranial neuropathies, papilledema, and myelopathy (2, 9). Children commonly present with seizures, developmental delay, increased intracranial pressure, and cognitive changes (10, 11). On examination, hemiparesis, ataxia, cranial neuropathies, altered mental status, tremor, and ataxia are observed (11). There are no classical symptoms or signs of GC owing to the extensive and unpredictable invasion of tumor cells into cerebral hemispheres and deep midline structures.

**Figure 1:**
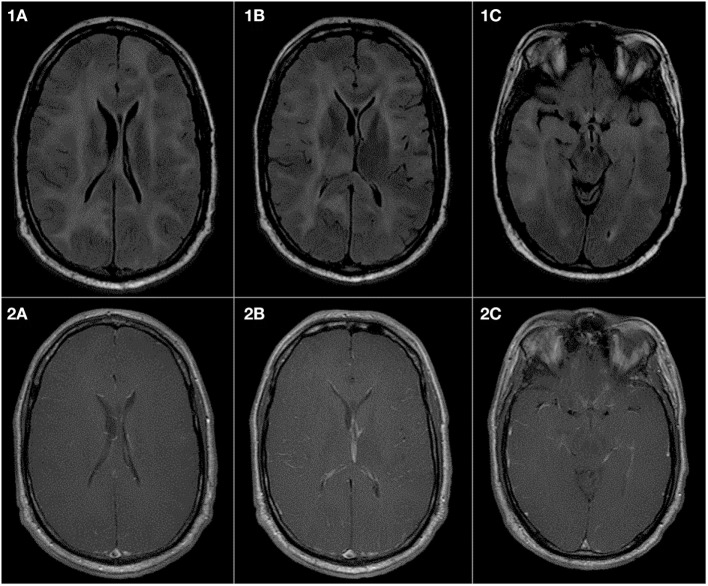
Gliomatosis cerebri presenting in a 43-year-old man as a syncopal episode, insidious onset of slurred speech and left leg weakness. Pathology was consistent with IDH-wildtype anaplastic astrocytoma. **(1A–1C)** are FLAIR sequences demonstrating extensive abnormalities in the white matter of both cerebral hemispheres presenting as high signal intensity. There is also an involvement of midbrain and the right thalamus. The right temporal lobe shows more extensive abnormality as compared to the left temporal lobe. **(2A–2C)** are T1 sequences postcontrast and show no abnormal increased enhancement in the brain parenchyma or in the meninges.

Before the magnetic resonance imaging (MRI) era, many patients with GC died without an established diagnosis and GC was determined at autopsy. Currently, GC is diagnosed radiographically by MRI along with histopathologic confirmation of an astrocytic process (1, 12, 13). Brain MRI shows a T1-weighted hypo- or iso-intensity and T2-weighted or FLAIR hyperintensity in the involved areas (Figure 1). There may be diffuse infiltration of the cortex, poor gray-white matter delineation, enlargement of affected cerebral structures and thickened gyri (14, 15). Enhancement patterns are variable, with focal, multifocal, or nodular gadolinium-enhancement in 16–56% patients (1, 5). Radiographic differential diagnoses include multiple sclerosis, progressive multifocal leukoencephalopathy, Behcet’s disease, ischemia, viral encephalitis, vasculitis, subacute sclerosing panencephalitis, ischemia, and other CNS inflammatory diseases (5, 12). In children, GC can be misdiagnosed as encephalitis, acute disseminated meningoencephalitis, idiopathic intracranial hypertension, acute disseminated encephalomyelitis, tubercular encephalitis, leukodystrophy and primary progressive multiple sclerosis (16, 17).

On MR spectroscopy (MRS), the choline (Cho)/creatine (Cr) ratio is increased and the N-acetylaspartate/Cr ratio is decreased, as observed in other malignant brain tumors (15, 19). MRS cannot reliably differentiate GC from encephalitis, demyelinating disease, progressive multifocal leukoencephalopathy, or hemorrhage (20, 21). Perfusion MR findings typically demonstrate lack of elevation of mean relative cerebral blood volume (22), corresponding to a relative lack of vascular angiogenesis. Fludeoxyglucose-positron emission tomography (FDG-PET) is not particularly useful for initial diagnosis as hypometabolism (23) or hypermetabolism (24) is seen in areas with infiltration; however, FDG-PET can be of value in following patients longitudinally for the extent of tumor involvement and treatment response assessment.

## Histopathology and Molecular Classification

**Figure 2:**
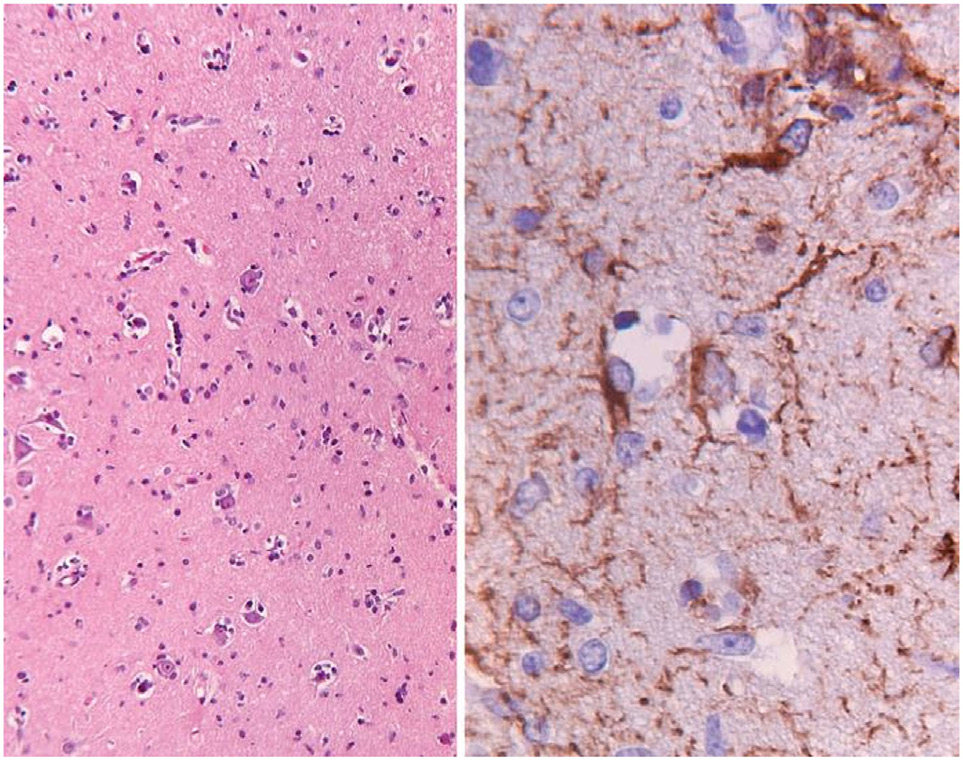
This is a pathology slide of a World Health Organization (WHO) grade II astrocytoma manifesting as gliomatosis cerebri. The left-hand panel shows hematoxylin–eosin-stained section of cortical tissue with a slight increase in cellularity (objective ×2.5). The right-hand panel shows GFAP staining for astrocytic cells (objective ×40), demonstrating somewhat irregular, atypical positive-staining cells. Buis et al. (18) © The Author(s) 2011. Reproduced with permission of Springer.

Most GC tumors are astrocytic, although oligodendroglial and mixed phenotype can rarely be seen. The gross anatomy remains intact, but affected areas appear firm, edematous, with flattened gyri and loss of gray-white distinction (25, 26). Though histological grading encompasses gliomas from grades II through IV, the clinical behavior of the tumor is consistent with an aggressive malignancy. GC classically has a diffuse, irregular parenchymal infiltration of glial cells, in contrast to the destructive, necrotic pattern seen in high-grade gliomas. Histologic exam reveals small, astrocytic cells with elongated fusiform nuclei, readily identified by staining for glial fibrillary acidic protein (Figure 2) (11, 25). In contrast to high-grade gliomas, neovascularization, significant mitotic activity and necrosis are not common (11). Because most tissue is obtained from a small biopsy specimen, the degree of intratumoral heterogeneity is unknown.

Our clinical and genetic understanding of many brain tumors are now refined by genomic studies and epigenome-wide methylation profiling, which have unraveled molecular subgroups in tumors such as glioblastoma, medulloblastoma, and ependymoma (27–30). However, application of these studies has not been insightful for GC. Surprisingly, molecular and methylation profiling showed that in both adults and children, there are no characteristic histologic features or molecular subgroups exclusive to this diagnosis. In a study of 25 adults with GC, patients were found to have isocitrate dehydrogenase (IDH) mutant astrocytoma, IDH mutant and 1p/19q codeleted oligodendroglioma or IDH wild type glioblastoma (RTK1, classic, mesenchymal, or H3F3A-G34 mutant) (1). Likewise, when Broniscer et al. analyzed 32 pediatric and adolescent patients with types I and II GC, their DNA methylation profile corresponded with known pediatric glioma molecular subgroups, including IDH mutant (17%), G34 (22%), mesenchymal (17%), and RTK I “PDGFRA” (44%) (13). All tumors were astrocytic and no codeletion of 1p and 19q was observed. No K27 mutation subgroup of pediatric high-grade glioma was identified, despite the fact that four patients had symmetrical bi-thalamic gliomas, which are typically associated with H3K27M mutation (29). As expected, molecular differences were seen between pediatric and adult GC; the IDH subgroup was less common and no oligodendroglioma or RTK II subgroup was observed in children (1, 13, 31).

## Treatment

There is no standard treatment for patients with GC. While a long indolent course and prolonged survival are rarely observed (32), the disease more typically progresses rapidly, with a median survival of <1 year in patients not receiving antitumor therapy (4, 5).

## Surgery and Tissue Acquisition

All patients in whom GC is radiographically suspected should have a histopathologic confirmation. Given the diffuse involvement of a large brain volume, the role of surgery primarily lies in securing a tissue diagnosis. Some patients undergo partial resection of an area of T2-signal abnormality or T1 contrast-enhancement to secure sufficient amount of tissue to overcome sampling error. When patients are symptomatic due to edema and mass effect, partial resection can be done with an aim of tumor debulking. It is unclear if extent of surgical resection provides any survival benefit. Perkins et al. reported outcomes in 30 GC patients of which 19 received biopsy and 11 had a partial resection (33). The median survival (21 versus 18 months, *p* value = 0.96) did not reach statistical significance.

## Radiation Therapy

The use of radiation therapy in GC is challenging due to the large-field involved and the apparent radioresistance of GC. Anecdotal evidence suggests stabilization of disease and resolution of neurological symptoms for a period of time in patients treated with radiation therapy alone (34–36). As GC histopathologically mirrors other gliomas, many institutions treat adult patients as high-grade glioma, with upfront radiation or chemo-radiation therapy. This approach raises concern in children given the large tumor volumes involved, the absence of a standard of care for children with high-grade gliomas, and the disputed evidence of efficacy of chemoradiation in pediatric malignant glioma (37). It is unclear whether radiation volume and/or dose correlates with outcome. Radiation therapy protocols have delivered radiation to involved field only, whole brain, or whole brain with a cone done to the involved field (11, 34). Whole brain radiotherapy doses ranging from 20 to 59 Gy (6, 7, 33, 34) and regional radiotherapy doses from 54 to 66 GY have been administered (6, 33). Chen et al. utilized a median radiation dose of 54.90 Gy and did not find any correlation between the total dose of radiation and survival (6). Four retrospective studies have reported a clinical response in 58% of patients and a radiographic response in 31% of patients (7, 33, 34, 38). Taillibert et al. reviewed a historic cohort of 296 patients and found that overall survival (OS) curves did not differ according to radiation treatment (*p* = 0.3) (5). In contrast, Chen et al. found the OS was significantly different (*p* < 0.01) in patients treated with (27.5 months) or without (6.5 months) radiation therapy (6). Despite clinical and radiographic improvement in many cases, response to radiation therapy is not durable and the evidence for its impact on OS is, at best, ambivalent.

## Chemotherapy

Patients with GC usually receive chemotherapy alone or in conjunction with radiation. However, no study has demonstrated significant efficacy of chemotherapy in this disease. Table 1 reviews chemotherapy data derived from historical cohorts in patients with GC. NOA-05 is the only prospective clinical trial published to analyze the efficacy of primary chemotherapy in GC (39). This study was a phase II single arm study in which 35 patients with GC were treated with procarbazine and lomustine as upfront therapy. The median progression-free survival (PFS) was 14 months and median OS was 30 months. Although it is difficult to draw conclusions about superiority of upfront radiation versus chemotherapy regimen when comparing results of this study to retrospective historical cohorts who received radiotherapy only (median OS 11.4–38.4 months) (7, 33, 34, 38), the NOA-05 trial results suggest that initial treatment with procarbazine and lomustine may have potential clinical benefit for patients with GC.

**Table 1:** Clinical studies reviewing treatment of patients with gliomatosis cerebri.

Reference	Study type	Treatment	No. of patients	Median PFS (months)	Median OS (months)	Histology (%)	Comments
Glas et al. (39), NOA-05	Prospective	PC	35	14	30	DA 20 (57) AA 7 (20) OA 2 (6) AO 2 (6) GB 4 (11)	MGMT in 12/25 (48%) IDH1 mutation in 12/25 (48%) PC in upfront setting

Glas et al. (40)	Retrospective	PC	12	16	37	OD 2 (17) DA 10 (83)	PC used in upfront setting

Sanson et al. (4)	Retrospective	PCV versus TMZ	17-PCV 46-TMZ	15.8 16 (no significant difference)	25.6 26.4 (no significant difference)	OD 43 (72) OA 6 (10) A 11 (18)	Both primary and secondary GC with no focal mass

Soffietti et al. (41), AINO	Retrospective	TMZ	46	9	14	DA 15 (33) AA 8 (17) OD 4 (9) AO 1 (2) OA 2 (4) GB 3 (7) Others 13 (28)	Both upfront and treatment at progression

Levin et al. (8)	Retrospective	TMZ	11	13	Not reached	DA 2 (18) OD 6 (55) AO 1 (9) OA 1 (9) GB 1 (9)	6 patients received PCV upfront Patients with GC with or without focal mass

Kaloshi et al. (42)	Retrospective	TMZ	25	18	37.7	Oligodendroglial 14 (56) Astrocytic 9 (36) Others 2 (8)	Primary and secondary GC Upfront treatment

*AA, anaplastic astrocytoma; AO, anaplastic oligodendroglioma; DA, diffuse astrocytoma; GB, glioblastoma; OA, oligoastrocytoma; OD, oligodendroglioma; PC, procarbazine and lomustine; TMZ, temozolomide*.

Temozolomide is widely used for treatment of adult malignant gliomas and is often used in treatment of GC. Samson et al. retrospectively compared response rate to procarbazine, vincristine and lomustine (PCV) versus temozolomide in a series of 63 patients with GC. No significant difference was observed in the PCV and temozolomide groups in PFS (15.8 versus 16 months) or OS (25.6 versus 26.4 months), but increased toxicity was noted in the PCV group. Retrospective studies have demonstrated that temozolomide can be used in the treatment of GC, both as initial therapy and at progression with a median PFS and OS ranging from 9 to 18 and 14 to 37.7 months, respectively (4, 8, 41, 42). A report from Levin et al. suggested that temozolomide may also be used after initial tumor progression with PCV treatment (8). Of 2 patients whose treatment was changed from PCV to temozolomide, one had continued disease progression but the other was stable for 12 months. Given the variability in PFS and OS in historical cohorts of patients with GC, randomized phase II studies may better elucidate the roles of chemotherapy in this disease.

Patients with GC who have oligodendroglial pathology and 1p/19q codeletions have a higher radiographic response rate, PFS, and OS when treated with temozolomide as compared to patients with non-oligodendroglial GC (4, 42). These data are not surprising given our current knowledge about the chemosensitive nature of oligodendroglial tumors and longer overall patient survival when compared with those with astrocytic tumors. Similar evidence of chemosensitivity can be found from some case reports and studies where nitrosourea-based regimens were used upfront (4, 39, 43, 44). From current literature, it appears that both temozolomide- and nitrosourea-based regimens may be useful for initial treatment of adult patients with oligodendroglial GC, yet no conclusion can be drawn about the superiority of one treatment over another.

As most cases of GC show a lack of contrast-enhancement on MRI and CT, neovascularization is considered to be rare or absent in this disease (45). In contrast to this assumption, a study found strong VEGF expression in five of six patients and COX2 expression in four of six patients despite the absence of contrast-enhancement on MRI. Additionally, histopathology and CD31 antibody studies demonstrated vascular proliferation in gliomatosis affected areas. Patients in this non-randomized study were treated with a combination of low-dose temozolomide and celecoxib and had PFS of 6–18 months (46).

While new treatment paradigms using immunotherapy are being developed for high-grade gliomas, these have not formally evaluated in patients with GC. Generally, tumor cells survive by dysregulating the body’s immune checkpoints by overexpressing immunosuppressive surface ligands such as programmed cell death-1 (PD-1) and cytotoxic lymphocyte-associated protein-4 (CTLA-4). Immune checkpoint inhibitors such as nivolumab (anti-PD-1), pembrolizumab (anti-PD-1), and ipilimumab (anti-CTLA-4) are being investigated for CNS tumors, including glioblastoma. With their success in various solid tumors like melanoma and non-small cell lung cancer (47, 48), they may be of potential benefit in a heterogeneous disease entity such as GC and thus need to be investigated. Additionally, clinical trials (NCT02746081) are underway to test IDH inhibitors in gliomas (49, 50) as the IDH mutation can be found in 17–48% of adults with GC (1, 13, 39). Little is known about metabolism and metabolic defects in GC. However, a major issue in evaluating efficacy of chemotherapeutic agents for GC patients is inconsistent inclusion in clinical trials, lack of GC-specific cohorts, and variable definitions of GC for eligibility.

## Challenges

The initial challenge in management of GC is timely diagnosis. Symptoms and MRI findings are non-specific, therefore a confirmed diagnosis of GC is delayed. When patients present with advanced symptoms and large tumor volumes, treatment options can be limited, although whether earlier diagnosis and treatment is associated with better outcomes is unknown. Because of the relatively extensive differential diagnosis, tissue confirmation is critical. However, biopsy results in only limited tissue available for diagnosis and molecular testing. Advanced molecular studies are necessary to further our understanding of GC and identify potential targets for therapy. Understanding the tumor pathophysiology and specifically the biology behind the extreme invasiveness of tumor cells is a first step toward developing novel therapeutics for GC. In the First International Gliomatosis cerebri Group Meeting held in Paris, France in March 2015, it was suggested that at least two different biopsy samples using the same needle tract at different depth be obtained to increase tissue sampling (16). Additionally, advanced imaging modalities like MRS, perfusion MR and FDG-PET along with standard MRI, should be incorporated in GC management as they can aid in delineating the extent of disease, selecting the appropriate surgical site for biopsy and in treatment assessment.

Given the lack of randomized controlled trials, it is unclear if radiation therapy or chemotherapy benefits patients with GC, if GC subtypes have different responses, or if pediatric and adult GC differ biologically. Only one prospective study, NOA-05, has been published to date and this was a non-randomized study using a historical cohort. Treatment conclusions gleaned from retrospective series are subject to publication bias and offer limited information due to small sample size. Randomized studies for radiation therapy are complicated by the heterogeneity of the disease. Additionally, large-field partial brain radiotherapy or whole brain radiotherapy may have a significant impact on quality of life.

Because of GC’s clinical, molecular and pathological heterogeneity, it is difficult to evaluate efficacy of a specific therapy as the study population and historical cohorts vary. Defining criteria for randomization is critical. Several studies have attempted to prognosticate survival and outcomes in retrospective analysis using clinical, radiographic and, more recently, molecular data. With our current knowledge that the molecular profiles of adult and pediatric GC are not distinct from glial tumors of corresponding WHO grades, the next pertinent question is whether grade and histology of the tumor plays any role at all in prognosis and prediction of outcome. While two studies with a relatively large sample size retrospectively analyzed the relationship of tumor grade and survival (5, 6), others did not find that tumor grade was prognostic of outcome (1, 4, 13, 39, 51). Recent data from genome-wide DNA methylation analysis on 25 patients have also shown that WHO grades were not prognostic of outcomes, but patients with a molecular classification of classic/RTK2 or mesenchymal glioblastoma fared worse (1). Sanson et al. showed that a pure oligodendroglial pathology of GC was associated with a significantly better outcome (4), but this was not validated by Glas et al., likely as his study included only four patients with oligodendroglial pathology, all of which were oligoastrocytomas (39). Various studies have reported an OS of 18–35 months for grade II, 12–29 months for grade III and 9–36 months for grade IV GC (1, 4, 5, 34, 51). Even though these data are highly variable, contrast this to OS of 8–13 years in grade II gliomas (52), 37 months to 15 years for grade III gliomas (53, 54), and 15–16 months in glioblastoma (55, 56). It is quite clear that patients with GC fare much worse than patients with diffuse gliomas of corresponding grades.

Knowledge about molecular profile has helped to refine prognostication in other adult diffuse gliomas. Mutations affecting the IDH genes 1 and 2 are associated with longer OS as compared to patients with IDH wild type genes, although this does not hold true for pediatric patients. The IDH mutation can be found in up to 48% of adult patients with GC (1, 40, 57), but there is no clear relationship with outcome (*p* = 0.08) (1) or to better prognosis (39, 40, 57, 58). Molecular data and its relationship with survival are scanty in children. To date, of 19 pediatric patients tested for IDH mutations in two different studies, only 3 (16%) patients had tumors with an IDH mutation (13, 31). MGMT promoter methylation is another molecular marker related to better outcomes in adults with malignant glioma (1, 39), but its applicability in children is unclear (13).

A number of studies looked at the association of age and outcome to determine if older age in adults is related to a poor outcome, but no convincing evidence is seen (4–6, 39, 51). In a small cohort of pediatric patients, Armstrong et al. found that age at diagnosis was a significant predictor of OS with children diagnosed in the first decade of life faring poorly (11), however, another pediatric study found no such relationship (13). Similarly, the relationship between good performance status and outcome is not consistent with studies reporting a better outcome with a high performance status (5, 6), while others failed to find an association (4, 39). No correlation is found with presenting neurological symptoms and outcome (6, 13). Most studies have found no relationship with the MRI appearance of lesion (contrast-enhancement, symmetrical presentation) with patient prognosis (1, 6, 11, 13, 39). However, children who present with symmetrical bi-thalamic involvement show a poor prognosis (13). Two studies have found that patients with substantial gray matter involvement (e.g., thickening of cortex, insula, basal ganglia and thalamus) have worse outcomes than patients with predominantly white matter involvement (diffuse swelling of hemisphere, swelling of corpus callosum, and loss of gray-white differentiation) (59, 60). The conclusions from these studies are limited by the relatively small numbers of patients.

## Conclusion

Gliomatosis cerebri remains a poorly understood entity that affects all age groups. Despite aggressive treatment, patients have a uniformly poor outcome. There are a paucity of studies evaluating the biology and pathophysiology of this disease. Although no longer considered a distinct pathologic entity, GC represents a disease with a unique phenotype. We now have the tools to increase our knowledge of the molecular biology of GC; further study of the biology driving this migratory and invasive growth pattern of tumor cells and its microenvironment is necessary to better define GC. These investigations may lead to new therapeutic targets or more rational therapeutic management of these patients.

## Author Contributions

Both authors jointly developed the design and arguments for the paper, drafted the manuscript, reviewed, approved the final manuscript, and are accountable for all aspects of the work.

## Conflict of Interest Statement

The authors declare that the research was conducted in the absence of any commercial or financial relationships that could be construed as a potential conflict of interest.
